# Chondroinduction of Mesenchymal Stem Cells on Cellulose-Silk Composite Nanofibrous Substrates: The Role of Substrate Elasticity

**DOI:** 10.3389/fbioe.2020.00197

**Published:** 2020-03-19

**Authors:** Runa Begum, Adam W. Perriman, Bo Su, Fabrizio Scarpa, Wael Kafienah

**Affiliations:** ^1^Faculty of Biomedical Sciences, School of Cellular and Molecular Medicine, University of Bristol, Bristol, United Kingdom; ^2^Bristol Dental School, University of Bristol, Bristol, United Kingdom; ^3^Bristol Composites Institute (ACCIS), University of Bristol, Bristol, United Kingdom

**Keywords:** cellulose, silk, electrospinning, mesenchymal stem cells, substrate elasticity, chondrogenesis

## Abstract

Smart biomaterials with an inherent capacity to elicit specific behaviors *in lieu* of biological prompts would be advantageous for regenerative medicine applications. In this work, we employ an electrospinning technique to model the *in vivo* nanofibrous extracellular matrix (ECM) of cartilage using a chondroinductive cellulose and silk polymer blend (75:25 ratio). This natural polymer composite is directly electrospun for the first time, into nanofibers without post-spun treatment, using a trifluoroacetic acid and acetic acid cosolvent system. Biocompatibility of the composite nanofibres with human mesenchymal stem cells (hMSCs) is demonstrated and its inherent capacity to direct chondrogenic stem cell differentiation, in the absence of stimulating growth factors, is confirmed. This chondrogenic stimulation could be countered biochemically using fibroblast growth factor-2, a growth factor used to enhance the proliferation of hMSCs. Furthermore, the potential mechanisms driving this chondroinduction at the cell-biomaterial interface is investigated. Composite substrates are fabricated as two-dimensional film surfaces and cultured with hMSCs in the presence of chemicals that interfere with their biochemical and mechanical signaling pathways. Preventing substrate surface elasticity transmission resulted in a significant downregulation of chondrogenic gene expression. Interference with the classical chondrogenic Smad2/3 phosphorylation pathway did not impact chondrogenesis. The results highlight the importance of substrate mechanical elasticity on hMSCs chondroinduction and its independence to known chondrogenic biochemical pathways. The newly fabricated scaffolds provide the foundation for designing a robust, self-inductive, and cost-effective biomimetic biomaterial for cartilage tissue engineering.

## Introduction

Smart biomaterials with an inherent capacity to control stem cell differentiation can have a significant impact on the field of cartilage tissue regeneration. Currently, the majority of studies demonstrating the successful use of biomaterials for cartilage tissue engineering require the addition of specific growth factors to induce chondrogenic differentiation and control stem cell fate (Meinel et al., 2004; Lee et al., 2006; Coleman et al., 2007; Kafienah et al., 2007a), which are often costly with limited efficacy. Eliminating the need for exogenous biological stimulation would not only reduce the costs of production, but also circumvent the adverse effects associated with such biologics; for example, intra-articular injections of transforming growth factor-β (TGF-β) result in osteophyte formation in the murine joint (Van Beuningen et al., 2000). The ideal biomaterial for cartilage tissue engineering would provide a number of attributes that closely align to that of the stem cell niche, which plays a critical role in maintaining stem cell function and fate; orchestrating a plethora of physical and chemical cues to regulate cell proliferation, differentiation, migration and apoptosis (Scadden, 2006; Martino et al., 2012). Currently, a range of biomaterial types are used for *in vitro* cartilage tissue engineering, including hydrogels, foams/sponges and functionalised bioceramics (Armiento et al., 2018; Freedman and Mooney, 2019).

Biomaterials used in tissue engineering act as a scaffold for cell growth *in vitro* and cell delivery *in vivo*, supporting the cells whilst they develop and establish their own *de novo* scaffold—the extracellular matrix (ECM). The *in vivo* ECM largely consists of high-strength fibrous collagens embedded in a hydrated proteoglycan matrix, allowing cell to cell communication and directed tissue formation. The collagen fibers, composed of nanometre-scale multifibrils, form 3D macroscopic tissue architectures, which vary between tissue types, with fiber diameters ranging from 50 to 500 nm (Muir et al., 1970; Elsdale and Bard, 1972; Ottani et al., 2001). Biomaterial fabrication processes have begun focusing on mimicking this nanoscale morphology, and electrospinning has been widely utilized for the development of 3D fibrous scaffolds, particularly in the field of cartilage tissue engineering (Li et al., 2005; Subramanian et al., 2005; Nerurkar et al., 2011; Shanmugasundaram et al., 2011; Garrigues et al., 2014; Kuo et al., 2014; Torricelli et al., 2014).

Electrospinning involves the fabrication of polymer fibers through the exploitation of electrostatic forces. Both natural and synthetic polymer sources have been employed in such technology, producing fibers that range in diameters from a few nanometres to several micrometers (Reneker and Chun, 1996). Electrospinning, therefore, allows the assembly of an artificial ECM retaining the cells native nano-structural milieu. Compared to other fiber spinning processes, electrospinning permits the generation of long fibers with smaller diameters and higher surface area-to-volume ratios. In the context of tissue engineering, fibrous materials would be advantageous to resident cells, by supporting the efficient exchange of nutrients, gases and waste products. It is not surprising therefore, that such a method has been widely explored for various applications, including but not limited to, skin, bone and blood vessels (Reneker and Chun, 1996; Pham et al., 2006; Ingavle and Leach, 2014).

Mechanical aspects of a cells external environment can influence its fate. For example, biomaterial elasticity has been identified as a driving factor in determining MSC lineage specification. Engler et al. demonstrated that hMSCs differentiate toward the lineage respective of the substrate elasticity upon which they are cultured (Engler et al., 2006). Soft matrices mimicking that of brain led to cells differentiating into a neuronal phenotype; cells seeded on stiffer matrices, as that seen in muscle displayed a myogenic phenotype; and that on rigid matrices, as in collagenous bone, became osteogenic. Beyond surface elasticity, several other scaffold-dependent morphological and chemical properties can influence stem cell differentiation. Addressable chemical functional groups on surfaces interacting with MSCs can direct their differentiation down specific lineages (Curran et al., 2005), perhaps through the activation of specific differentiation signaling pathways. Curran et al. showed that when MSCs were seeded on silane-treated glass surfaces functionalised with hydroxyl (-OH) or amide (-NH_2_) groups, the cells expressed chondrogenic and osteogenic mRNA respectively, in the absence of stimulating factors (Curran et al., 2005). The nanoscale topography of a biomaterial may also influence stem cell fate. Dalby et al. demonstrated how the structural organization of an *in vitro* matrix can influence MSC differentiation (Dalby et al., 2007). Cells grown on semi-disordered substrates expressed calcifying bone proteins, whereas those seeded on flat substrates showed no such induction.

Herein we describe, for the first time, the direct electrospinning of cellulose-silk in a 75:25 mass ratio using a TFA-AcOH cosolvent system. We demonstrate the biocompatibility of these novel composite nanofibres with hMSCs and reveal a peculiar relationship between the level of chondroinduction and media FGF-2 concentration. Furthermore, in line with our previous work (Singh et al., 2013), we fabricate the composite materials as cast films and begin to unravel the biophysical signaling mechanisms at the cell—biomaterial interface. We reveal the significant role of substrate elasticity in driving the chondroinduction seen.

## Materials and Methods

### Electrospun Nanofibres

Bombyx mori silk (Aurora Silk) was degummed to obtain silk fibroin fibers as follows; fibers were cut into 1 cm pieces and boiled in 0.02 M Na_2_CO_3_ solution, followed by washing then left to dry. Cellulose from wood pulp (DP 890), was purchased as sheets (Rayonier Inc.) and ground to a powder. Trifluoroacetic acid (TFA, 99%) and glacial acetic acid (AcOH, ≥99.85%) were purchased from Sigma Aldrich. A 4 wt% concentration blend solution of cellulose:silk in a 75:25 ratio was prepared in TFA. The polymers and solvent were placed in a glass vial containing a stir bar, sealed and placed on a magnetic stirrer at room temperature (RT) for 8 days. Following this time period, AcOH was added to the solution at 20% (v/v) and electrospun immediately. A horizontal electrospinning setup was used under a fume hood and set-up as follows; the solution was loaded into a syringe (5 ml Leur-LokTM, Beckton Dickinson), and a stainless steel blunt-end 22-gauge needle attached (Precision glide, Beckton Dickinson). This was placed onto a syringe pump (PHD 2000 Infusion Pump, Harvard Apparatus), facing a flat metal plate covered with aluminum foil. A high voltage power supply (EL Series 1–30 kV, Glassman High Voltage Inc) was connected to the needle tip and grounded at the metal plate (collector) with a tip-to-target distance of 10 cm. A 4 wt% concentration solution was electrospun at 1.0 ml/hr flow rate and 2.0 kV/cm voltage. All experiments were performed at RT. Environmental temperature and relative humidity (RH) were also monitored (Testo 608-H2 Humidity, Temperature & Dewpoint Hygrometer 120603). The AcOH-TFA solvents were removed through evaporation during the electrospinning process. The materials were chemically characterized to confirm this (see below).

### Cast Films

Cellulose and silk were prepared as discussed above. Composite films of cellulose:silk in a 75:25 ratio were prepared as before (Singh et al., 2013). Briefly, polymers were weighed to prepare a 1.5% concentration solution in 5 g of 1-ethyl-3-methylimidazolium acetate (EMIMAc, Sigma Aldrich) which was then heated to 85°C with stirring, for 2 h. Polymer solutions were then poured into pre-heated glass dishes and left to cool overnight. Ethanol:acetic acid (EtOH:AcOH 90:10, respectively) was then added to aid coagulation of the polymers. Samples were covered and left overnight. Any remaining solvent was then removed by immersing in distilled water. The 2D films were then dried on parafilm.

### Material Characterization

#### Electrospun Nanofibres

Fiber morphology and diameter was assessed using a combination of scanning electron microscopy (SEM) and ImageJ software (Schneider et al., 2012). Fibrous mats were placed on a carbon pad and coated with gold in argon, with a plasma current of 18 mA for 15 s (SC7620 Mini Sputter Coater, Quorum Technologies), then imaged using a field emission gun SEM (Zeiss EVO Series SEM, Carl Zeiss) at 15 kV accelerating voltage and 24 mm working distance. Micrographs were loaded into ImageJ and fiber diameter/beading measured (ImageJ 1.x). Chemical characterization was performed on the nanofibrous mats using Fourier transform infrared spectroscopy (Spectrum 100 FTIR spectrometer, PerkinElmer). Analysis was performed in transmission mode across a spectral range of 4,000–700 cm^−1^. Spectra were generated three times per sample.

#### Cast Films

Composite films were also imaged using SEM. Samples were prepared and imaged as above. Chemical characterization was also performed on cast films, as above, using FTIR.

### hMSC Culture

hMSCs were isolated from human bone marrow plugs recovered from patients undergoing complete hip replacement arthroplasty, with their informed consent. Sample collection was carried out following local ethical guidelines in Southmead Hospital, North Bristol Trust. Cells were characeterised and isolated following our established protocol (Kafienah et al., 2006) Following isolation, hMSCs were expanded in stem cell expansion medium consisting of low glucose Dulbecco‘s modified Eagles medium (DMEM), 10% (v/v) fetal bovine serum (FBS, Thermo Scientific Hyclone), 1% (v/v) Glutamax (Sigma), and 10% (v/v) penicillin (100 units/mL)/streptomycin (100 mg/ml) antibiotic mixture (P/S, Sigma). Media was supplemented with 5 or 10 ng/ml fibroblast growth factor 2 (FGF- 2, PeproTech). Cells were cultured at a density of 2 × 10^5^ cells per cm^2^ and incubated at 37°C in a humidified atmosphere of 5% CO_2_ and 95% air. Media was changed every 2–3 days and cells were passaged upon reaching 80–90% confluency. Cells used for all experiments were between passage 1 and 5.

### Cell Loading on Composite Materials

#### Electrospun Nanofibres

Composite nanofibrous mats were cut into 8 mm discs using a biopsy punch (Stiefel, Schuco Intl). Discs were disinfected by immersing in aqueous 70% ethanol (v/v) for 30 min and then washing with phosphate buffered saline (PBS). For fibronectin coated scaffolds; following the wash step, PBS was removed and 100 μg/ml human plasma fibronectin in PBS was added (Sigma). Adsorption of fibronectin on polymer surfaces enhances cell adhesion (Kowalczyn et al., 2001). For non-fibronectin coated scaffolds; following the wash step, PBS was removed, and scaffolds immersed in further PBS. Scaffolds were then incubated overnight in PBS. Following incubation, scaffolds were transferred to 24-well ultra-low attachment plates (Corning, Costar, Sigma). Plates were covered and scaffolds left to dry (~2 h). Expansion media supplemented with 5 or 10 ng/ml FGF- 2 was then added to each well and cells plated at a density of 28 × 10^3^ cells per cm^2^ on scaffolds and tissue culture plastic (TCP) controls. As a positive control for chondrogenesis, hMSCs were cultured on tissue culture plastic in the presence of transforming growth factor β (TGFβ) (Figure S5). This soluble growth factor is a potent stimulator of chondrogenesis in MSCs (Barry et al., 2001). Media was changed every 2–3 days.

#### Cast Films

Composite substrates were sterilized, and fibronectin coated as discussed above. hMSCs were also plated at a density of 28 × 10^3^ cells per cm^2^ on cast films and TCP controls.

### Characterizing hMSC Behavior on Composite Materials

#### Electrospun Nanofibres

hMSC metabolic activity on nanofibrous mats was assessed using Alamar Blue (AB) assay (ThermoFisher). The AB dye is a non-toxic fluorescent dye that undergoes a REDOX reduction in the presence of cellular growth, fluorescing and changing color (Ansar Ahmed et al., 1994). The AB assay was performed at days 1, 3, and 7 following initial cell loading (day 0) on the same wells. hMSCs were cultured on the nanofibrous surfaces in expansion medium supplemented with 5 ng/ml FGF-2. AB dye reduction was quantified using a spectrophotometer at 560 nm and 600 nm wavelengths (GloMax-Multi+ Microplate Multimode Reader with Instinct, Promega) and percentage reduction calculated following a standard protocol (alamarBlue® protocol, Bio-Rad). Following the AB assay at day 7, hMSC adhesion and viability was assessed using the Live/Dead Viability/Cytotoxicity assay kit (Invitrogen), following the manufacturer's protocol. Cells were incubated for 30 min at RT with 2 μM Calcein AM and 4 μM Ethidium homodimer-1. Cells were viewed under a widefield microscope (Leica DMIRB inverted microscope). hMSC morphology was visualized on the nanofibrous composites using SEM. Cell-loaded materials were fixed in 4% paraformaldehyde (PFA) then dehydrated in a series of ethanol solutions of increasing concentration. The samples were then dried using a critical point dryer (Leica EM CPD300) and prepared for SEM as discussed above.

#### Gene Expression Studies

hMSCs were cultured on the nanofibrous surfaces in the presence of 5 or 10 ng/ml FGF-2. RNA extraction was performed after 2 weeks using the RNeasy Plus Mini Kit (Qiagen) and PureLink RNA Mini Kit (Thermo Fisher Scientific), respectively, following manufacturer's instructions. RNA concentration was determined spectrometrically at 260 and 280 nm wavelengths (NanoPhotometer P-class Spectrophotometer, GeneFlow). 20 ng/ml of RNA elution was reverse transcribed to produce complementary deoxyribonucleic acid (cDNA) using the High Capacity cDNA Reverse Transcription Kit (Applied Biosystems) according to the manufacturer's protocol. Chondrogenic gene expression was quantified using polymerase chain reaction (qPCR, StepOne Plus, Life Technologies). qPCR was performed on 96-well plates with each sample in triplicate. Each well-contained 1 μl cDNA, 3.5 μl nuclease-free water, 0.5 μl TaqMan® Gene Assay primer and 5 μl TaqMan® Gene Expression Master Mix. The following TaqMan® Gene Expression Assays were used; Collagen type II alpha 1 (Col2), Aggrecan (Agg), SRY-box 9 (Sox-9), Collagen type I alpha 1 (Col1), Alkaline phosphatase (ALPL), Peroxisome Proliferator Activated Receptor Gamma (PPARG), and the housekeeping gene glyceraldehyde 3-phosphate dehydrogenase (GAPDH) all purchased from Life Technologies Ltd. Data was analyzed using the double delta Ct analysis method. Gene expression levels were normalized to the expression levels of the housekeeping gene and presented as fold change relative to control conditions; cells in the same media conditions cultured on tissue culture plastic.

### Cast Films

#### Gene Expression Studies

Composite substrates were cultured with hMSCs in stem cell expansion media supplemented with 10 ng/ml FGF-2, as per the media conditions used previously (Singh et al., 2013). Downstream gene analysis was performed as described above.

#### Substrate Elasticity and Chondrogenic Signaling of hMSCs on Cast Films

To evaluate the cell morphological response to substrate elasticity, cells were fluorescently stained for paxillin (a focal adhesion protein) and their cytoskeleton (using a phallotoxin that binds to filamentous actin). Briefly, samples were washed with PBS then permeabilised at RT with 0.1% Triton X-100 for 10 min. Following a further wash step, the appropriate primary antibody was added; Anti-PXN rabbit antibody (5 μg/ml, Sigma Aldrich) or normal rabbit IgG control antibody (5 μg/ml, LifeTechnologies). Samples were then covered and left overnight at 4°C. Following incubation, samples were washed, and secondary antibodies added; Alexa-Fluor 594 donkey anti-rabbit IgG (5 μg/ml, LifeTechnologies) and FITC-conjugated Phalloidin (2 μg/ml, Sigma Aldrich). Samples were covered and left at RT for 1 h. Samples were then washed and a DAPI stain (NucBlue® Fixed Cell ReadyProbes™ Reagent, LifeTechnologies) added and incubated for a further 15 min. The DAPI stain was then removed and samples washed and left in PBS until imaging.

Blebbistatin (Sigma), a NMMII inhibitor, was used to investigate the impact of substrate elasticity on stem cell differentiation potential. The toxicity and potency of the inhibitor when cultured with hMSCs was established in our lab and a concentration of 1 μM was used, in line with published values (Das et al., 2013). The toxicity and potency of the TGF-β type I receptor kinase inhibitor, SB505124 (Sigma) when cultured with hMSCs was also established (Tang et al., 2009). A concentration of 1 μM was identified as least toxic with maximum inhibition (data not shown).

### Statistical Analysis

hMSCs were used from five patients (*n* = 5) on electrospun materials and from 4 patients (*n* = 4) on cast film materials. Statistical significance was assessed using the two-way ANOVA with Bonferroni post-test. *p* ≤ 0.05 was taken as significant. Data points on graphs show mean ± standard error of the mean (SEM).

## Results and Discussion

### Electrospinning Cellulose-Silk Composite Nanofibres

**Figure 1:**
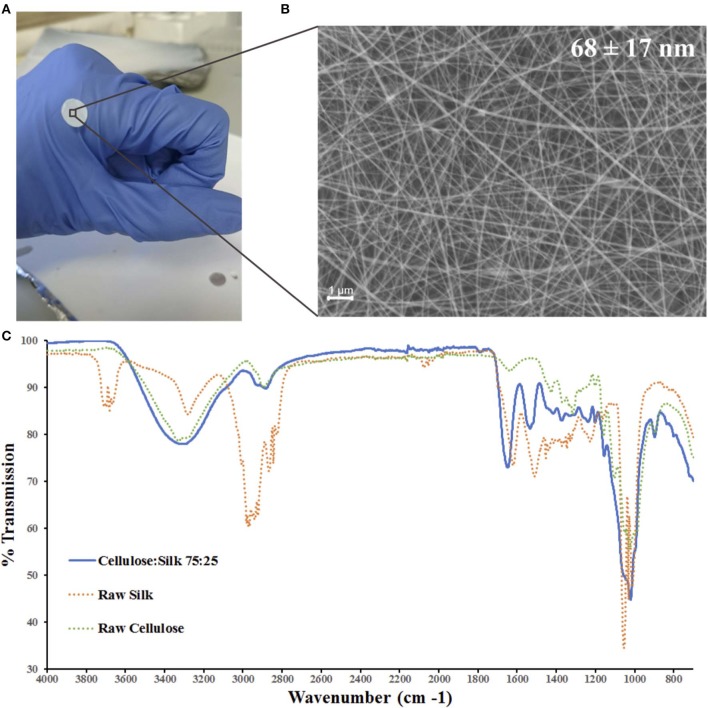
Electrospun cellulose:silk 75:25 composite nanofibres. Polymer solutions were electrospun at 1.0 ml/h flow rate and 2.0 kV/cm voltage in a trifluoroacetic acid—acetic acid (TFA+AcOH) co-solvent system. **(A)** 8 mm discs were cut for cell culture studies. **(B)** Scanning electron microscopy of the nanofibrous material with fiber diameters of 68 ± 17 nm (*scale bar inset measures 1* μ*m*). **(C)** FTIR was performed to chemically characterize the nanofibrous mat. Solid line—electrospun fibers, dashed line—raw polymers. Key shown inset.

Cellulose and silk blend solutions were prepared in both TFA only and TFA-AcOH cosolvent systems at a range of concentrations, and electrospun at various operational parameters. A 4 wt% concentration solution of the blended polymers in the cosolvent system electrospun at a 1.0 ml/hr flow rate and 2.0 kV/cm voltage was identified as ideal, generating a continuous electrohydrodynamic jet and resulting in the deposition of uniform nanofibres (Figures 1A,B). Further investigations revealed that the morphology of the nanofibres was affected by the environmental conditions, specifically relative humidity (RH) (Figure S1). Environmental temperature and RH were not controlled during the electrospinning experiments; however, they were recorded. The former parameter remained largely within a narrow range on all occasions, suggesting RH was playing the principal role in the significant differences in fiber diameter and morphology seen (Figure S2). As RH increased, fiber diameters decreased, whilst bead length, width and frequency increased. Both cellulose and silk polymers are insoluble in water. An increase in RH reflects an increase in the amount of water vapor present in the air. Thus, the polymers precipitate faster, limiting elongational and thinning forces resulting in larger fiber diameters (De Vrieze et al., 2009). Furthermore, during electrospinning fiber beading can result due to increased jet instability in the whipping phase of the spinning process. Specifically, net charge density, surface tension and solution viscosity can influence the level of this instability (Jaeger et al., 1998; Fong et al., 1999; Zuo et al., 2005; Liu et al., 2008). The results seen here are likely a combination of these competing factors at play. Environmental temperature remained within a narrow range in all instances (Figure S2).

Chemical characterization of the nanofibrous composite was performed using FTIR (Figure 1C). The regenerated polymers retained their native chemical functionalities. A strong broad band in the 3,600–3,000 cm^−1^ region denoting the O-H bond stretching of cellulose was present as was a narrower band at 2,850–2,920 cm^−1^, characteristic of asymmetric and symmetric stretching of methyl and methylene groups in cellulose, respectively (Garside and Wyeth, 2003; Popescu et al., 2011; Poletto et al., 2014). The three characteristic regions of the silk fibroin peptide backbone are referred to as amide I (1,700–1,600 cm^−1^), amide II (1,600–1,500 cm^−1^), and amide III (1,350–1,200 cm^−1^) (Miyazawa and Blout, 1961; Barth, 2000; Barth and Zscherp, 2002). Strong absorption bands are seen in all three regions for the composite fibers (Figure 1C). The spectra of the composite polymer material showed broader bands at higher frequencies relative to the raw polymers. This can be attributed to changes in inter- and intramolecular hydrogen bonding, both within the protein and polysaccharide (Miyazawa and Blout, 1961; Garside and Wyeth, 2003; Poletto et al., 2014) as well as between the protein and polysaccharide (Freddi et al., 1995; Yang et al., 2000, 2002). Critically, the characteristic absorption bands of the solvents used—TFA and AcOH—were absent (Max and Chapados, 2004; Valenti et al., 2011).

### Human Mesenchymal Stem Cell Viability on Composite Nanofibres

Nanofibrous mats with zero to minimal fiber beading were used in cell studies (≤ 20 beads per 100 μm^2^; Figure S2B). No significant difference was noted in cellular read-outs with/without the presence of beads and so materials were used interchangeably. hMSCs were cultured on the composite nanofibres with and without fibronectin (FN) coating. Fibronectin is an ECM glycoprotein involved in cell adhesion; enabling communication between intracellular and extracellular environments via cell surface integrin receptors (Ruoslahti, 1984; Loeser, 1993). It is commonly used in biomaterial cell cultures to enhance cell adhesion to natural/synthetic material surfaces (Kowalczyn et al., 2001; Harnett et al., 2007; Franck et al., 2013; Agarwal et al., 2015; Jacobsen et al., 2017). The use of this adhesion protein does not conceal the surface functional groups of neat and composite cellulose-silk cast films, retaining their chemical influence on hMSC behavior (Singh et al., 2013).

**Figure 2:**
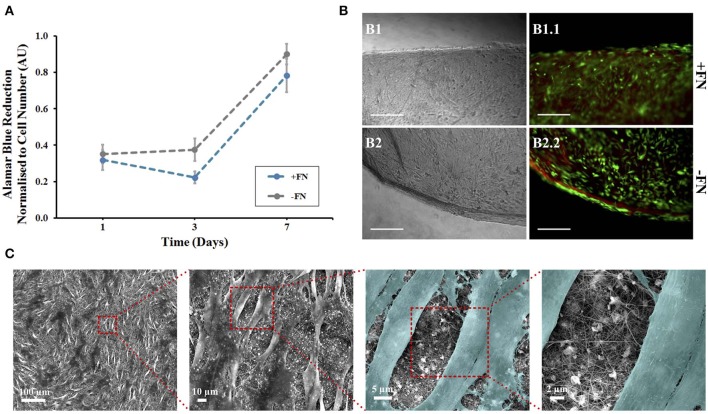
Human mesenchymal stem cells on composite electrospun nanofibres. Cells were cultured on nanofibrous discs in the presence of 5 ng/ml FGF-2 with (blue line) and without (gray line) fibronectin (FN) coating. **(A)** Viability at 1, 3, and 7 days using Alamar Blue assay, data points show mean ± SE. **(B)** A LIVE/DEAD viability assay was performed at day 7. Optical (B1,B2) and fluorescence (B1.1,B2.2) microscopy images of cells on composite nanofibres. Live and dead cells show green and red fluorescence, respectively. Scale bar inset measures 300 nm. **(C)** Samples were then fixed and viewed using scanning electron microscopy. Cells were colored in latter images to enhance contrast between cells and nanofibres (*n* = 5).

Alamar blue (AB) assay was used to quantitatively assess hMSC viability and metabolic activity on the nanofibrous mats, with and without FN coating (Figure 2A). The AB assay was performed on the nanofibrous mats and tissue culture plastic control samples at three time points—day 1, 3, and 7 following initial cell culture (day 0). Dye reduction was extrapolated to deduce cell number based on a standard curve (Figure S3B) and results normalized. hMSCs were loaded at the same density on both surface types. Cells cultured on control plastic surfaces remained viable and showed a gradual increase in metabolic activity over the 7 day period (Figure S3A). hMSCs cultured on nanofibrous mats maintained similar viability over the 7 day period, with and without FN coating (Figure 2A). This result can be understood by considering the inherent chemical nature of the materials. Indeed, biomaterial surface chemistry can impact MSC adherence, differentiation and protein adsorption *in vitro*. The characteristic functional groups of the natural polymers used here—hydroxyl (-OH) for cellulose and amine (-NH_2_) for silk—have been widely studied, with their impact on MSC behavior characterized (Keselowsky et al., 2003; Curran et al., 2005, 2006; Hao et al., 2016). Both -OH and -NH_2_ groups encourage MSC adhesion and proliferation through the promotion of cell surface integrins (Hao et al., 2016), with the latter group demonstrating a higher affinity (Curran et al., 2005). Material surface chemistry can also influence the conformation of adsorbed proteins. Surface chemistry influences the conformation of adsorbed FN, and therefore it's cell binding affinity, *via* the exposure of specific surface integrins (Keselowsky et al., 2003). Both -OH and -NH_2_ functionalised surfaces with adsorbed FN direct its conformation such that cell binding is enhanced. Furthermore, the strength of cell binding is greater on -OH functionalised surfaces (Keselowsky et al., 2003). However, it is also important to state that there was statistically no significant difference between hMSC metabolic activity on nanofibres with and without this fibronectin coating (Figure 2A) and no visible difference in cell binding (Figure 2B). It can therefore be concluded that use of this adhesive protein does not mask the surface functional groups of these natural polymers, as has been previously confirmed using these polymers (Singh et al., 2013), and therefore, their respective roles in cell binding affinity. Furthermore, composite blends of cellulose and silk have demonstrated an increase in surface hydrophilicity relative to their native counterparts (Feng et al., 2017) and this can positively influence cellular adhesion and proliferation (Leal-Egaña et al., 2013). The composites inherent hydrophilicity may be the cause of the cellular response seen, regardless of fibronectin coating. Although electrospun polymer fibers often demonstrate a reduction in hydrophilicity relative to their cast films (Wang and Wang, 2012; Feng et al., 2017), with smaller diameter fibers showing higher contact angles (Cui et al., 2008), both cellulose and silk electrospun nanofibers have shown hydrophilic behavior (Rodríguez et al., 2014; Mohammadzadehmoghadam and Dong, 2019). The TFA-AcOH cosolvent system used in their fabrication did not elicit any detrimental effects, as anticipated from the chemical characterization performed on the materials (Figure 1C). Regardless of FN coating on the nanofibres, the reduction of AB (and therefore cellular metabolic activity) increased over the 7 day period, demonstrating not only the biocompatibility of these materials but also their ability to support cellular proliferation.

The non-toxic nature of the AB assay enabled qualitative analysis to be performed on the same samples to visualize cell adherence on nanofibrous and plastic surfaces. Viable cells were viewed on all surfaces, corresponding to the AB assay results at day 7 (Figure 2B). The hMSCs grown on tissue culture plastic, as a positive control, demonstrated a spindle morphology, characteristic of healthy undifferentiated hMSCs (Figure S4A). The presence of dead cells was negligible. hMSCs killed with methanol, used as a negative control, all showed red fluorescence demonstrating their compromised membrane integrity, with loss of their spread spindle morphology (Figure S4B). For hMSCs grown on the composite nanofibres, images were taken at the scaffold edge and center to visualize whether cell spread was uniform across the 8 mm disc. In all cases, cells were evenly spread across the nanofibrous surface, regardless of FN coating. Cell morphology was more compact than cells grown on plastic with negligible dead cells (Figure 2B).

In order to evaluate cell morphology at a higher resolution on the composite nanofibres, following 7 days of *in vitro* culture the cell-laden biomaterials were fixed and viewed under SEM (Figure 2C*, image shown of FN-coated surface*). Densely populated cell layers with clear cell-to-cell contacts were seen on the materials, regardless of FN coating. Critically, the fibers had retained their nanofibrous morphology and the mats retained their overall shape and integrity until the end of the *in vitro* culture.

### Human Mesenchymal Stem Cell Chondroinduction on Composite Nanofibres

Having established hMSC viability and proliferation on these nanofibrous materials, and therefore their biocompatibility, the stimulative capacity of the scaffold was investigated. hMSCs were cultured on FN-coated nanofibrous composites in the absence of stimulating factors. Cells were also grown on tissue culture plastic under the same media conditions as control. Cells were cultured in 5 ng/ml and 10 ng/ml FGF-2. FGF-2 is used in hMSC culture *in vitro* due to its ability to enhance cellular proliferation and maintain stem cell differentiation potential (Martin et al., 1997; Solchaga et al., 2005). It is not known to behave as a chondrogenic stimulator. To confirm the specific chondrogenic differentiation of hMSCs on the composite biomaterial, gene expression for other potential lineages was measured. This included the osteogenic marker (ALPL) and the adipogenic marker (PPARG) (*data not shown*).

**Figure 3:**
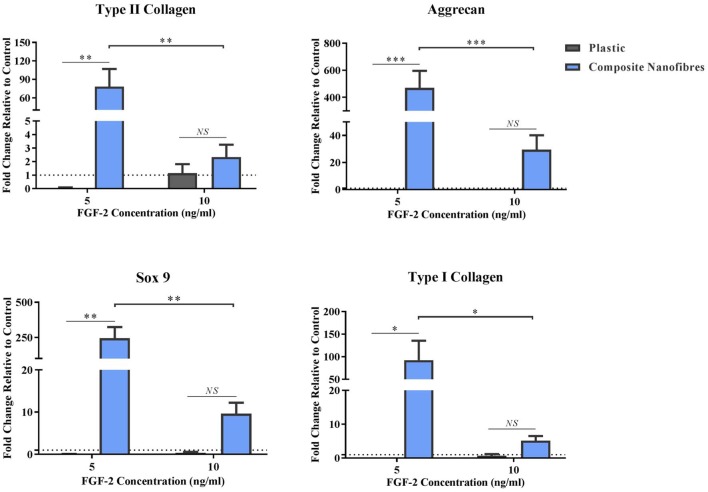
Stimulative capacity of composite nanofibrous mats. hMSCs were cultured on plastic and composite nanofibres in the absence of soluble chondrogenic factors. Following 14 days culture, qPCR was performed to quantify the level of chondrogenic gene expression—Col2, Agg, and Sox-9—as well as Col1. Gene expression levels were normalized to the expression of the housekeeping gene. GAPDH (shown in dotted line). Graph shows mean ± SE. Samples were compared statistically using two-way ANOVA with Bonferroni *post-test*. *p < 0.05, **p < 0.01, ***p < 0.001, *n* = 5.

hMSCs on FN-coated nanofibrous and plastic surfaces maintained viability over the longer investigation period (14 days). Following the culture period, downstream analysis was performed to screen for the expression of chondrogenic genes on all surfaces (Figure 3). The expression levels of type-II collagen (Col-2), aggrecan (Agg) and SRY-box 9 (Sox-9) were assessed, as key markers for chondrogenesis in hMSCs (Zhao et al., 1997; DeLise et al., 2000; Kiani et al., 2002; Kolf et al., 2007). Type-I collagen levels were also assessed as a marker unusually associated with hMSC chondrogenic differentiation *in vitro*; more commonly associated with hMSC dedifferentiation (Roberts, 1985; Mayne, 1989; Marlovits et al., 2004). Expression levels of all genes were normalized to that of the housekeeping gene GAPDH.

All chondrogenic genes studied on the nanofibrous composites were upregulated, relative to the hMSCs control grown on plastic (Figure 3). This upregulation was statistically significant at the lower FGF-2 concentration of 5 ng/ml. These results strongly indicate that the nanofibrous composite surface can influence hMSC behavior and direct its differentiation without the need for stimulating factors. Importantly, the significance of gene upregulation is influenced by the concentration of FGF-2. The latter is likely due to the hMSCs being under the influence of a higher proliferative stimulus, thereby overriding hMSC capacity to differentiate. At the lower FGF-2 concentration however, the inherent material characteristic driving hMSC differentiation appears to overtake the FGF-2 proliferative effect. Taken together, these results demonstrate the potent capacity of the nanofibrous cellulose/silk composites to drive chondrogenic differentiation independent of soluble chondrogenic growth factors, making these composites exciting candidates for tissue engineering applications. Here, the introduction of a biomimetic nanofibrous matrix resulted in a more potent impact on chondroinduction than that previously reported on cast films of the same composition (Singh et al., 2013).

The impact of electrospun fiber diameter on cell growth and differentiation *in vitro* is well-established (Li et al., 2006; Wise et al., 2009; Shanmugasundaram et al., 2011; Hsia and Corbett, 2012; Noriega et al., 2012; Bean and Tuan, 2015; Pelipenko et al., 2015). Electrospun fibers of nano- and micro- scale appear to support chondrogenic differentiation (in the presence of stimulating factors) in a context dependant manner (Li et al., 2006; Wise et al., 2009; Shanmugasundaram et al., 2011; Noriega et al., 2012; Bean and Tuan, 2015). hMSCs cultured in the presence of stimulating factors show a preference for micro- scale fibers when undergoing chondrogenesis (Shanmugasundaram et al., 2011; Bean and Tuan, 2015), whereas nano- scale fibers are favored by chondrocytes—the terminally differentiated cells exclusive to cartilage tissue (Li et al., 2006; Noriega et al., 2012). It is important that the specific inherent material characteristic implicated in driving this chondroinduction is understood. This will not only assist future biomaterial design considerations, but also introduce the possibility to control and tune the level of gene expression induced. To this avail, we fabricated cast film composites of the polymers, as reported previously (Singh et al., 2013), and began to unveil the potential mechanisms driving this behavior at the cell-biomaterial interface. The 2D-cast films were chosen for such investigations to eliminate any role that a 3D nano-scale material may have on cell behavior.

### The Chondrogenic Signaling Pathway and hMSC Chondrogenesis on Composite Films

Cast film composite substrates were prepared as discs of 8 mm diameter (Figure S6) and characterized. The cast films demonstrated chondrogenic autoinductive potency through upregulation of chondrogenic genes and deposition of key ECM proteins, following culture with hMSCs.

We hypothesized that the specific composition of cellulose and silk, in a 75:25 ratio, with its distinctive chemical functionalities (-OH and -NH_2_, respectively), is responsible for the stimulation of chondrogenesis in hMSCs (Curran et al., 2005; Singh et al., 2013). To investigate this potential impact, we investigated the principal biochemical chondrogenic differentiation signaling pathway—TGFβ-driven Smad2/3 phosphorylation (Johnstone et al., 1998). Phosphorylation of the Smad2/3 protein complex results in its translocation to the cell nucleus where it can regulate transcription factors and drive the upregulation of chondrogenic genes. Stimulation via this pathway leads to the upregulation of Sox9 which activates the expression of type II collagen genes (Kamachi et al., 2000; Furumatsu et al., 2005; Haudenschild et al., 2010). This process occurs independent of substrate elasticity (Park et al., 2011) and can be selectively inhibited using the small molecule inhibitor SB505124, a TGFβ type I receptor kinase inhibitor (DaCosta Byfield et al., 2004).

**Figure 4:**
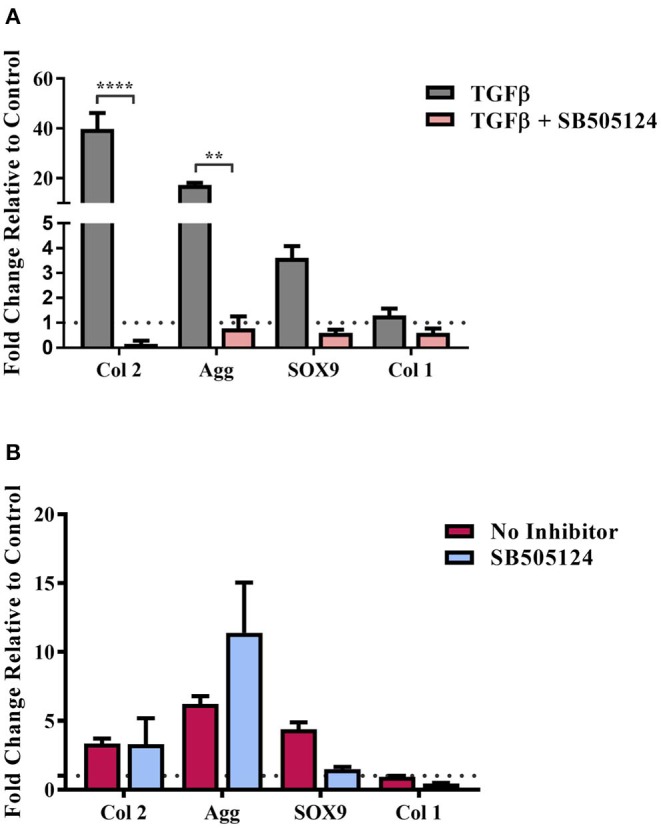
TGFβ-driven Smad2/3 phosphorylation and stem cell chondrogenesis. hMSCs were cultured on **(A)** Tissue cultured plastic in chondrogenic media supplemented with transforming growth factor-β (TGFβ) and **(B)** composite cellulose:silk 75:25 substrates in stem cell expansion media supplemented with 10 ng/ml fibroblast growth factor 2, with and without the SB505124 inhibitor. Cells were cultured for 14 days after which gene analysis was performed. Gene expression levels were normalized to expression levels of housekeeping gene, GAPDH (shown in dotted line). Data points show mean ± SE. Statistical significance was assessed using a two-way ANOVA with Bonferroni *post-test*. **p < 0.01, ****p < 0.0001, *n* = 4.

hMSCs were seeded on the composite substrates in the presence of SB505124 and gene expression levels were assessed 14 days later (Figure 4). As a control, hMSCs were seeded on plastic in the presence of TGFβ (Figure 4A). Addition of SB505124 to hMSCs on plastic in the presence of TGFβ resulted in a significant down regulation in type II collagen and aggrecan genes with a reduction in Sox9 expression also noted; demonstrating the potency of the inhibitor (Figure 4A). When the inhibitor was added to composite substrate cultures, there was no statistical difference between treatment and control for the expression of the chondrogenic markers Sox9, aggrecan, or type II collagen. The expression of type I collagen, a fibrochondrogenic marker, was also unchanged (Figure 4B). The lack of inhibition in hMSC chondrogenesis on the natural polymer composites in the presence of SB505124 suggests the chondroinduction caused by the composite occurs independently to TGFβ Smad2/3 phosphorylation. Whilst this phosphorylation is regarded as the principal signaling mechanism for hMSC chondrogenesis, it is important to consider other signaling pathways that may result in the same downstream effects on gene transcription. RhoA and Rho Kinase (ROCK) signaling can also stimulate the upregulation of Sox9 transcription resulting in the downstream expression of chondrogenic genes (Haudenschild et al., 2010). This pathway can be stimulated by TGFβ or mechanical stimulation (Haudenschild et al., 2010; Allen et al., 2012). In the latter instance, conducive substrates have been shown to exert a specific pressure at which ROCK activity is optimal for cells to undergo chondrogenesis and results in autocrine TGFβ production (Allen et al., 2012). This could suggest that the composite material is exerting the appropriate substrate stiffness to drive chondrogenic differentiation of the hMSCs and would explain the lack of significant down regulation in chondrogenic gene expression (Figure 4B).

### Substrate Elasticity and hMSC Chondrogenesis on Composite Films

**Figure 5:**
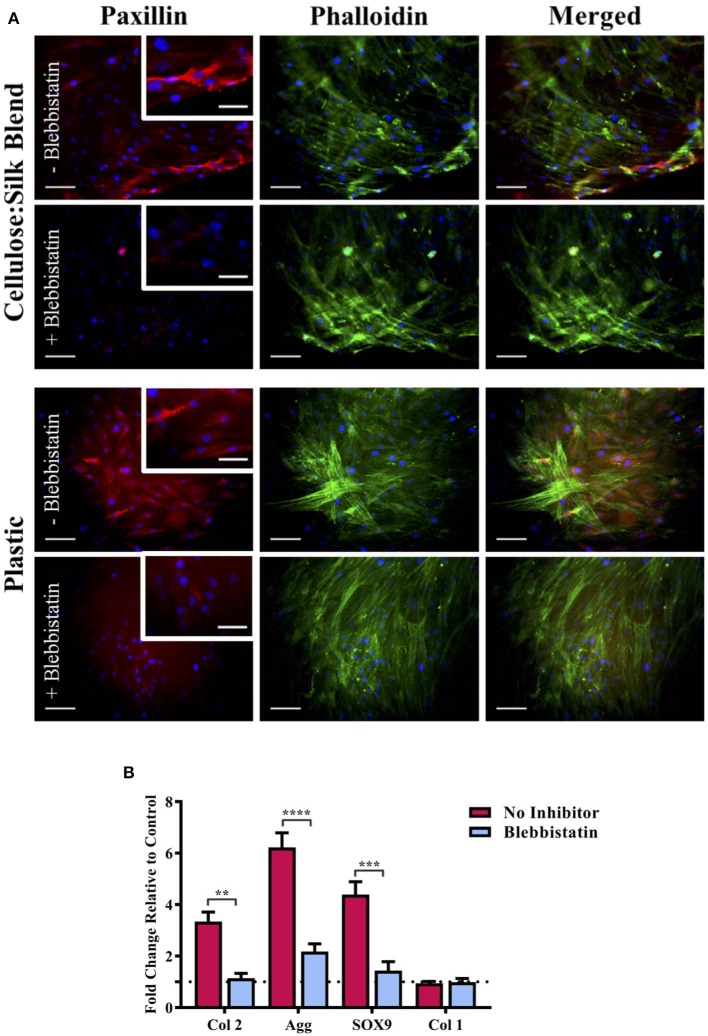
Substrate elasticity and stem cell chondrogenesis. hMSCs were cultured on cellulose:silk 75:25 composite substrates and tissue culture plastic in standard stem cell media supplemented with 10 ng/ml FGF-2. **(A)** Following 3 days culture, cells were fluorescently stained for their focal adhesions (paxillin, red) and cytoskeleton (phalloidin, green) to assess the physical response of cells to their substrate with and without the use of blabbistatin. Cell nuclei are stained using DAPI (blue). Scale bar inset measures 100 μm. Higher magnification images shown for paxillin staining inset, scale bar measures 50 μm. **(B)** Following 14 days of cell culture, gene analysis was performed. Gene expression levels were normalized to expression levels of housekeeping gene, GAPDH (shown in dotted line). Data points show mean ± SE. Statistical differences in gene expression were assessed using a two-way ANOVA with Bonferroni *post-test*. **p < 0.01, ***p < 0.001, ****p < 0.0001, *n* = 4.

We have previously demonstrated that cellulose and silk blended in a 50:50 ratio and neat cellulose films, do not have the same chondroinductive effect on hMSCs as cellulose-silk blended in a 75:25 ratio (Singh et al., 2013). Mammalian cells are known to respond to their substrate stiffness in both the number of adhesions formed to the surface and cell stretch (Pelham and Wang, 1997; Engler et al., 2006) and consequently in their differentiation (Engler et al., 2006). Cells on stiff substrates form a greater number of adhesions with increased cytoskeletal elongation compared to cells on more elastic substrates. During embryonic development, focal adhesion formation and integrin signaling are crucial for mesenchymal condensation and chondrogenic differentiation to take place (Loeser, 2014; Song and Park, 2014), however, *in vitro* culture of *ex vivo* chondrocytes has shown increased focal adhesion assembly to correlate with chondrocyte dedifferentiation (Shin et al., 2016). This discrepancy is further compounded by the evidence that mesenchymal stem cell chondrogenesis is encouraged *in vitro* by preventing focal adhesion attachment (Mathieu and Loboa, 2012); reflecting the complexity in defining the role of synthetic *in vitro* environments in line with our understanding of the *in vivo* environment. To investigate whether the mechanical properties of the composite can influence hMSC response to the substrate, the nature of hMSC adhesion and morphology was investigated. hMSCs were seeded on plastic in the presence of biochemical chondroinductive medium (10 ng/ml TGFβ, Figure S7) or without TGFβ (Figure 5A) as a negative control. Cells were cultured under appropriate media conditions with and without blebbistatin, a potent inhibitor of NMM II, which is a component of the cell actin cytoskeleton important in force transmission between intra- and extra-cellular environments (Straight et al., 2003). This selective inhibitor of NMM II ATPase has been shown to directly inhibit matrix elasticity-driven differentiation in hMSCs (Engler et al., 2006). Following 3 days in culture, cells were stained for the actin cytoskeleton and focal adhesion protein, paxillin. Cells in chondroinductive medium grown on plastic had a polygonal, rounded shape, as expected of hMSCs undergoing chondrogenic differentiation when stimulated with TGFβ (Figure S7) (Gao et al., 2010). The addition of blebbistatin reduced paxillin focal adhesion staining in all cases, suggesting a reduction in cell binding (Figure 5A) (Pelham and Wang, 1997; Engler et al., 2006). Cells grown on plastic in the absence of TGFβ showed elongated intense actin cytoskeletal structure and defined red fluorescent staining for the adhesion protein paxillin (Figure 5A). Cells cultured on the composite substrate showed a lesser cell stretch with diffuse paxillin staining, demonstrating the hMSC response to the elasticity of this surface (Figure 5A). These cells, however, did not demonstrate the distinct polygonal shape of hMSCs undergoing chondrogenesis (Figure S7), suggesting that the chondroinduction is partial and independent of the TGFβ signaling pathway. This finding further supports the results seen in the presence of SB505124 (Figure 4) and suggests that the potential mechanism for the chondroinduction of hMSCs grown on the cellulose-silk composites is dependent on surface elasticity.

To quantify the impact of elasticity-driven chondroinduction of these natural polymer composites, chondrogenic gene expression levels were investigated for hMSCs grown on the substrates in the presence and absence of blebbistatin (Figure 5B). Inhibition of the NMM II cytoskeletal protein by blebbistatin resulted in a significant downregulation in the expression of chondrogenic markers (Figure 5B). The expression of type I collagen, a marker of undifferentiated hMSCs, was not affected. Inhibition of the NMM II cytoskeletal protein complex has been specifically implicated in preventing substrate elasticity driven differentiation in hMSCs (Engler et al., 2006) and our results support this finding. It is worth noting that blebbistatin did not fully downregulate the chondrogenic markers to basal levels of the untreated control. This suggests that other signaling pathways that mediate cellular mechanotransduction may be implicated. The mechanical properties of this composite may support the induction of autocrine TGFβ production via ROCK signaling in conjunction with the NMM II mediated differentiation (Allen et al., 2012).

## Conclusion

Cellulose and silk composite nanofibres have been directly electrospun in a 75:25 mass ratio for the first time, with no post-spun treatments required. The morphology of these novel nanomaterials could be tuned through adjustments in operational and environmental parameters. Their biocompatibility has been demonstrated using hMSCs, supporting not only cell metabolic activity but also the ability to drive chondrogenic differentiation, without the need for soluble stimulating factors. Currently, research in the field of cartilage tissue engineering using biomaterials requires the use of stimulating factors to drive the chondrogenic differentiation of MSCs (Meinel et al., 2004; Lee et al., 2006; Coleman et al., 2007; Dalby et al., 2007; Kafienah et al., 2007b). Such stimulants are often costly, have limited efficiency, are short-lived and lead to terminal differentiation. These limitations beg the case for smart biomaterials with an inherent instructive capacity.

We have demonstrated the successful reproduction of a chondroinductive natural polymer composite into a more biomimetic configuration. Using the native ECM architecture to guide our biomaterial design, we have fabricated biocompatible nanofibrous networks with an inherent capacity to drive stem cell differentiation. It is important to highlight however that the electrospun biomaterials clearly lack sufficient porosity to aid cell infiltration (Figure 2C). Indeed, this is a common challenge when employing an electrospinning fiber fabrication technique. Ensuring porosity is sufficient to support cell infiltration and spread is key to developing an adequate material for tissue engineering applications and future design considerations would need to address this limitation. It remains to be investigated whether the level of chondrogenic marker genes and the detected ECM compares to established 3D pellet cultures, the gold standard for chondrogenesis studies. This would require fabricating the composite into a 3D configuration to provide the spatiotemporal signals associated with 3D cultures. Furthermore, these nanofibrous composites have the potential to be chemically modified with specific biomimetic chemical moieties to enable a more tailored cellular response (Gorgieva et al., 2017). The work presented here was performed under static media conditions, however the significance and importance of dynamic cell culture is widely reported. Particularly with reference to cartilage tissue, where conditions such as dynamic compression can enhance the level of non-hypertrophic chondrogenesis and improve the quality of engineered cartilage (Choi et al., 2018).

The potential inherent material mechanisms driving the chondroinduction of hMSCs were investigated on composite cast film substrates. The chondroinduction did not involve the principal chondrogenic differentiation signaling pathway—Smad2/3 regulated phosphorylation via TGF-β signaling. However, inhibition of force transmission between intracellular and extracellular environments via NMM II significantly reduced the chondrogenic gene expression of hMSCs on these substrates. The chondroinduction of hMSC seen on the nanofibrous surfaces of the same polymer composite are likely to also be driven by substrate elasticity. It is also important to note that although we have investigated the potential impacts of material chemistry and elasticity, we have not studied the impact of surface topography on cell behavior. The level of topographical disorder on a culture surface can impact hMSC differentiation, in the absence of stimulating factors (Dalby et al., 2007). We have previously assessed the surface topography of cast films demonstrating that increasing silk content of cast cellulose films results in a decrease in surface disorder, highlighting the impact of blending these polymers at the nano-level (Singh et al., 2013). Controlling for material topography, whilst retaining surface elasticity and chemistry on these natural polymer composites could prove particularly challenging. Further investigations are needed to reveal the interoperation of signaling pathways that orchestrate the transduction of physical cues into biochemical cues to mediate this differentiation.

## Data Availability Statement

The datasets generated for this study are available on request to the corresponding author.

## Author Contributions

RB developed the electrospun composite system, designed and performed the experiments, and wrote the manuscript. BS supported the electrospinning work. AP and FS contributed to the experimental design. WK conceived the original idea, supervised the project, and wrote the manuscript. All authors provided critical feedback and helped shape the research, analysis and manuscript.

### Conflict of Interest

The authors declare that the research was conducted in the absence of any commercial or financial relationships that could be construed as a potential conflict of interest.
