# Supplementary material for: Chondroinduction of Mesenchymal Stem Cells on Cellulose-Silk Composite Nanofibrous Substrates: The Role of Substrate Elasticity
Source: Front Bioeng Biotechnol. 2020 Mar 19;8:197. doi: 10.3389/fbioe.2020.00197 (PMC7096586; doi:10.3389/fbioe.2020.00197)
Supplement: Supplementary file 1 [file Data_Sheet_1.docx]

Supplementary Material

**Materials and Methods**

**Material Characterisation**

**Scanning Electron Microscopy:** For each testing condition, 10 SEM micrographs were taken. Fibre diameters were recorded by measuring distance across the width of fibres. Ten fibres were measured from each image, totalling 100 measured fibres per fibre type. Fibre beading was characterised in two ways; bead size and bead frequency. Bead size was characterised by measuring length (along the axis of the fibre) and width (across centre of bead). Five beads were measured from four different micrographs, totalling 20 measured beads per fibre type. Bead frequency was measured by isolating a 100 μm^2^ area (10 μm x 10 μm) of the micrograph and counting the number of beads enclosed. This was done on a total of four separate areas taken from different micrographs.

**Characterising hMSC Behaviour on Composite Materials**

**Electrospun Nanofibres**

**Gene Expression Studies:** Nanofibrous discs were cultured with cells in stem cell expansion media supplemented with 5 or 10 ng/ml FGF-2. Tissue culture plastic controls were also included as follows; hMSCs were cultured in standard 24-well tissue culture plates in stem cell expansion media supplemented with 5 ng/ml or 10 ng/ml FGF-2. hMSCs were also cultured on plastic in chondrogenic differentiation media as a positive control for chondrogenesis. The chondrogenic differentiation medium consisted of high glucose DMEM containing 1 mM sodium pyruvate, 1 % ITS, 1 % P/S, 50 μg/ml ascorbic acid-2-phosphate and 1x10^-7^ M dexamethasone (all from Sigma). This was supplemented with 10 ng/ml transforming growth factor-3 (TGF-β3, R&D Systems). A further control was also included – hMSCs cultured in chondrogenic differentiation medium without TGF-β3. Cells were cultured for 14 days before downstream gene analysis.

**Cast Films**

**ECM Protein Deposition:** Following 21 days culture of hMSCs on the composite substrate, ECM protein deposition was assessed using immunohistochemistry. Briefly, cells were washed with PBS then incubated for 20 minutes in 4 % PFA at RT. Cells were then washed again and permeabilised in 0.1 % Triton X-100. Following a further PBS wash step, the samples were incubated with goat anti-human aggrecan antibody (10 µg/ml, R&D Systems), goat anti-human Col2A1 antibody (4 µg/ml, Life Technologies) or normal goat IgG (control, 10 µg/ml) overnight at 2-8 ºC. Following incubation, samples were washed and then incubated with a fluorescent secondary antibody – donkey anti-goat Alexa Fluor® for one hour at RT (5 µg/ml, 594/488, Red/Green) (Life Technologies). Control samples (hMSCs grown on plastic under the same culture conditions) were also stained following the same protocol. Samples were imaged under a widefield microscope as before.

**Supplementary Figures**


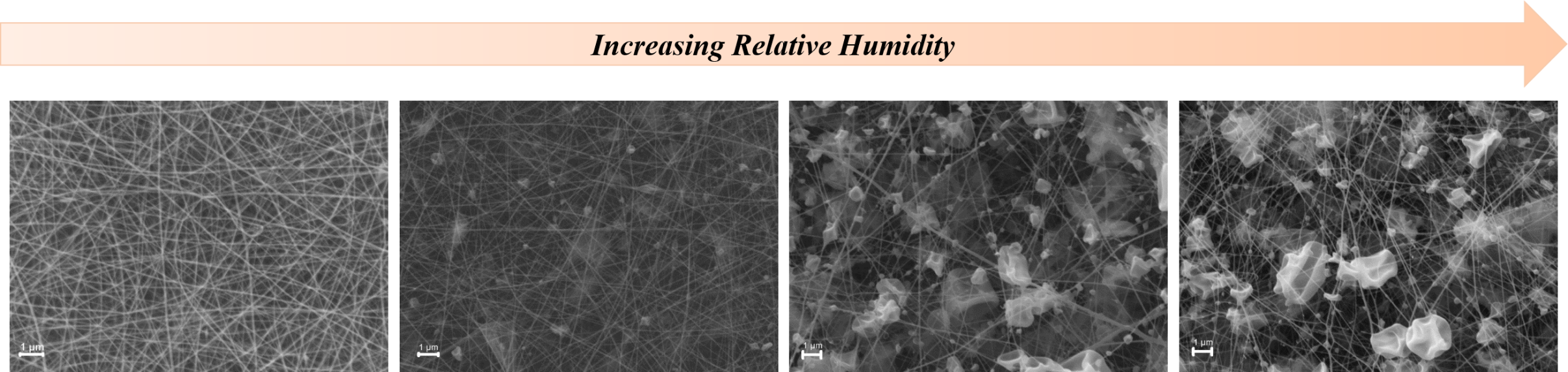


Figure S1. Electrospun Cellulose:Silk 75:25 Composite Nanofibres – Impact of Relative Humidity. Polymer solutions were electrospun at 1.0 ml/hr flow rate and 2.0 kV/cm voltage in a trifluoroacetic acid – acetic acid (TFA+AcOH) co-solvent system. An increase in environmental relative humidity affected fibre morphology. Scale bar inset measures 1 µm


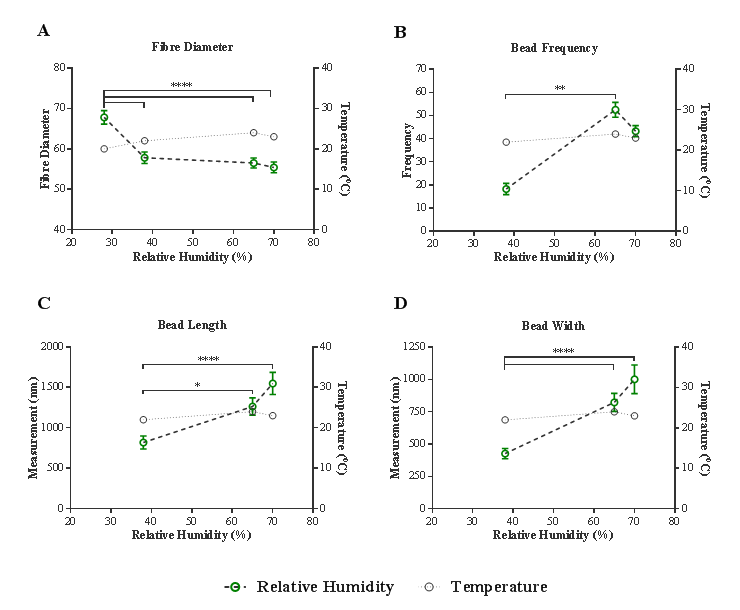


**Figure S2.** **Cellulose:Silk 75:25 Composite Nanofibres – Environmental Parameters and Fibre Morphology.** Polymer solutions were electrospun at 1.0 ml/hr flow rate and 2.0 kV/cm voltage in a trifluoroacetic acid – acetic acid (TFA-AcOH) cosolvent system. Graphs show impact of environmental humidity on A) Fibre Diameter, B) Bead Frequency, C) Bead Length and D) Bead Width. Fibre analysis shows mean of 100 measured fibres. Bead frequency analysis shows mean of four separate areas, each measuring 100 µm^2^. Bead analysis shows mean of 20 measured beads. All graphs show Mean ± SE (Left y axis, green). Non-parametric Kruskal-Wallis test with Dunn`s test post hoc applied to all graphs. *p*≤0.05 taken as significant. Environmental temperature remained within a narrow range across all samples (Right y axis, grey).

Figure S3. Human Mesenchymal Stem Cells on Plastic. hMSCs were cultured on tissue culture plastic (TCP) A) at 28x10^3^ cells per cm^2^ and an Alamar Blue assay performed at days 1, 3 and 7 and B) at a range of cell densities and an Alamar Blue assay performed 24 hrs later to generate a standard curve. Data points show mean ± SE, n = 5.


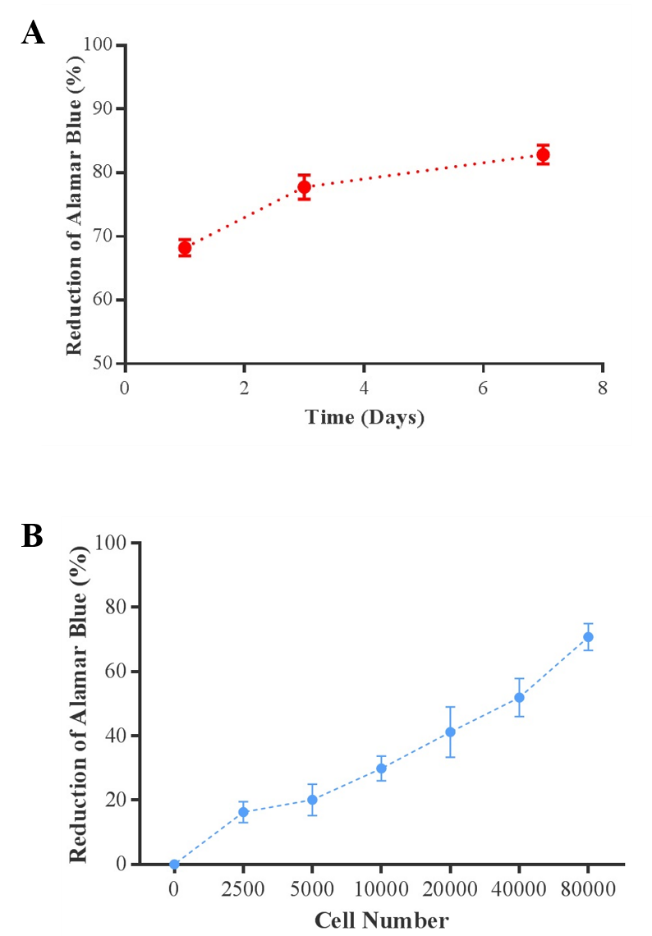


**Figure S4. Viability of Human Mesenchymal Stem Cells on Plastic.** Cells were grown on tissue culture plastic and their viability assessed after 7 Days using a LIVE/DEAD viability assay. Cells were either A), Ai) maintained in standard culture medium or B), Bi) killed with methanol, as a negative control, prior to staining. A), B), Optical microscopy and A.i), B.i) Fluorescence microscopy images of cells. Scale bar inset measures 300 nm. Representative images shown of cells from five patients. Live and dead cells show green and red fluorescence respectively.


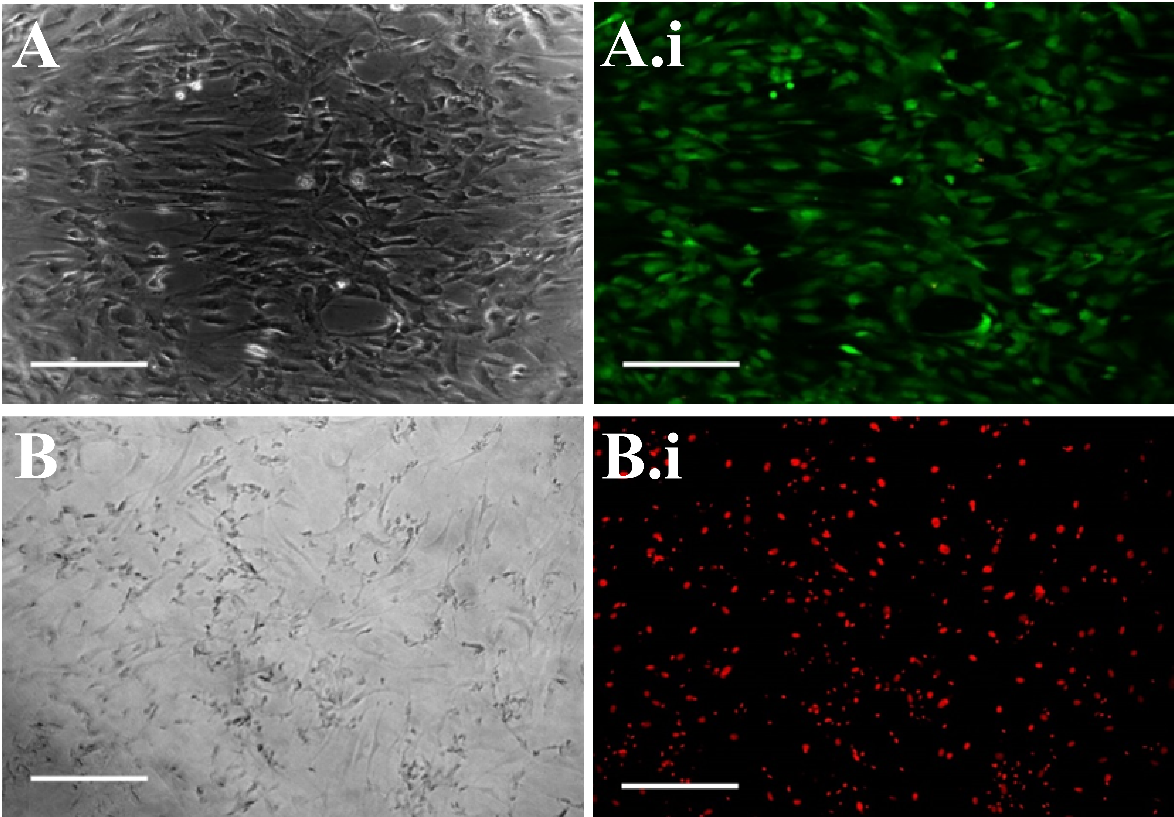

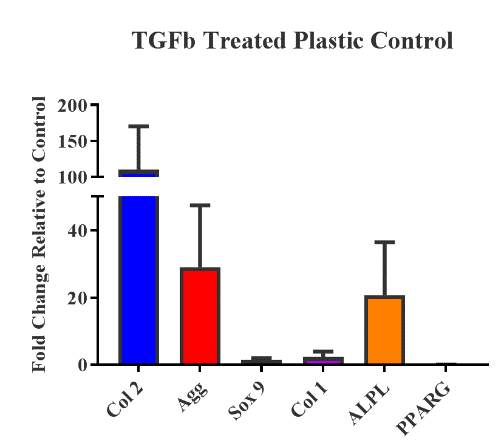


Figure S5. Human Mesenchymal Stem Cell Chondroinduction on Plastic. Cells were cultured on plastic in the presence of chondroinductive media. Cells were screened for the expression of key chondrogenic (Col 2, Agg, Sox 9, Col 1), osteogenic (ALPL) and adipogenic (PPARG) genes 14 days later. Gene expression levels were normalised to the expression of the housekeeping gene, GAPDH (shown in dotted line). Graph shows mean ± SE, n=5.


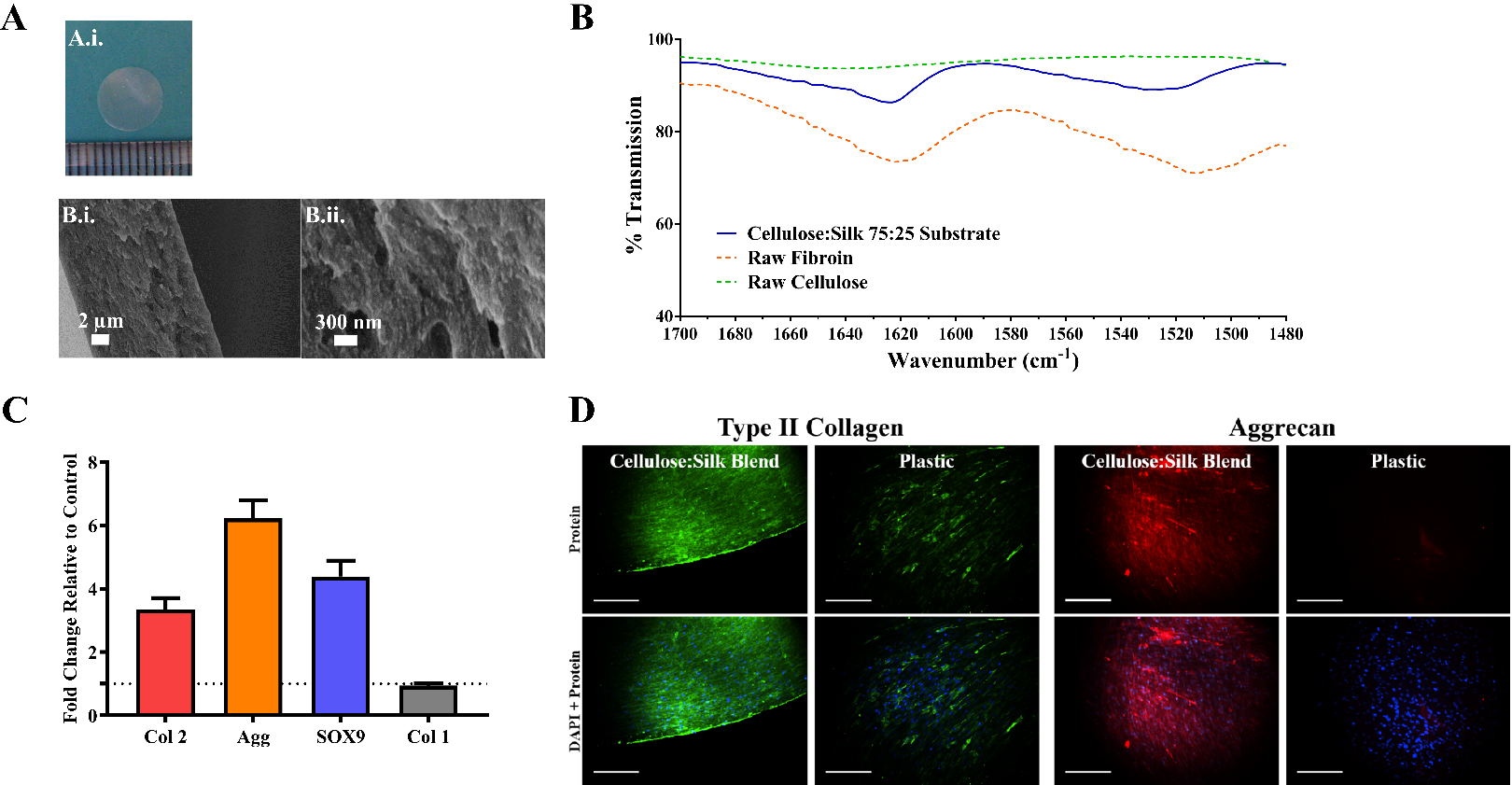


Figure S6. Human Mesenchymal Stem Cells on Composite Cast Film Substrates. The natural polymers were blended in a 75:25 ratio and cast films produced. A) Photo taken of an 8 mm disc of composite substrate and SEM of film cross-section, B) FTIR was performed on the regenerated cellulose:silk composite substrates (solid line) and the raw cellulose and silk polymers (dashed lines), C) hMSCs were cultured on fibronectin coated composite substrates in stem cell expansion medium supplemented with 10 ng/ml FGF-2. Following 14 days, cells were screened for the expression of the chondrogenic genes - Type II collagen (Col2), Aggrecan (Agg), Sox9 and the marker for dedifferentiation Type I collagen (Col1). Gene expression levels were normalised to the expression of the GAPDH housekeeping gene (shown in dotted line). Graph shows mean ± SE, D) Cells were also cultured for 21 days after which ECM protein deposition was assessed for key cartilage proteins – Type II Collagen (green fluorescence) and Aggrecan (red fluorescence). Cell nuclei were stained using DAPI (blue). Scale bar inset measures 300 µm, n=4.


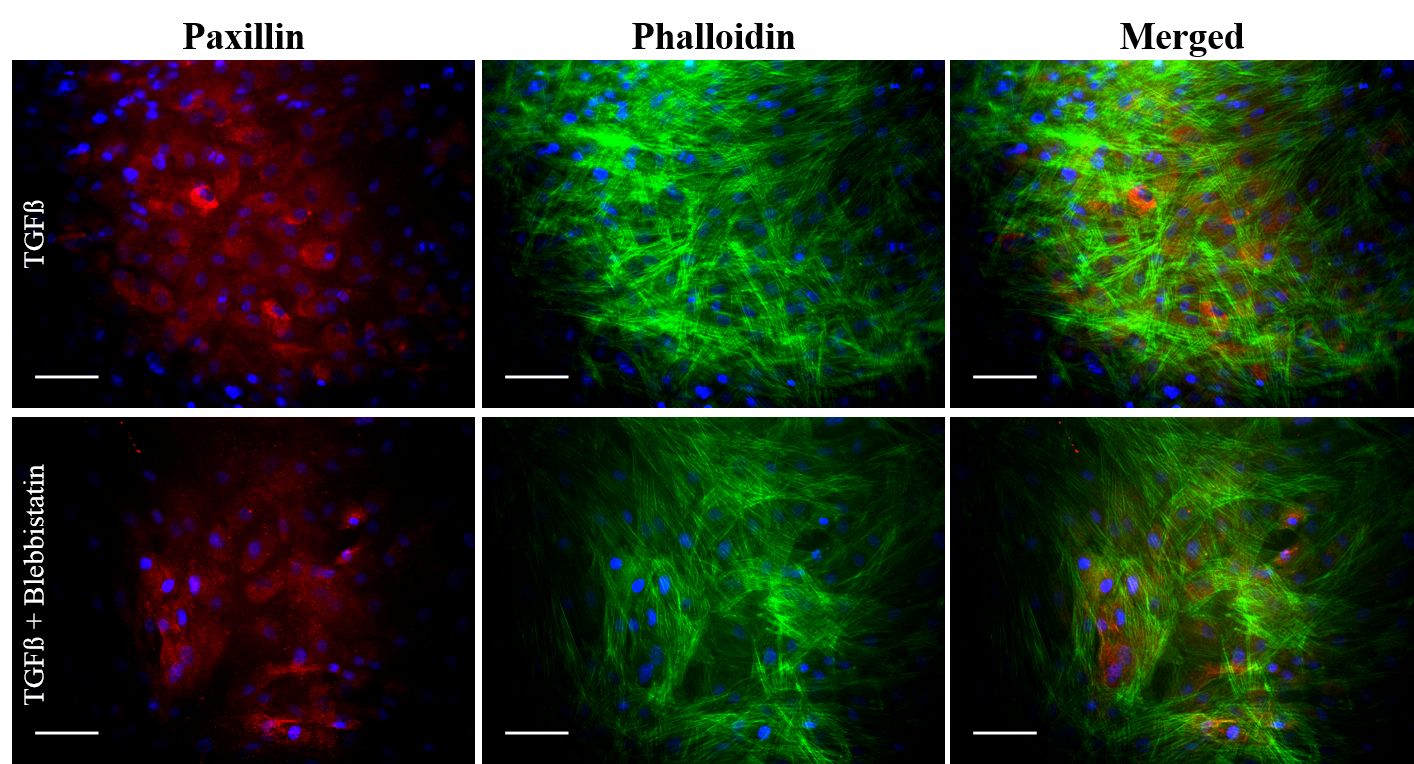

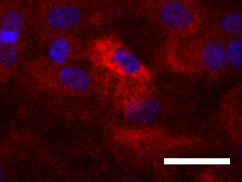

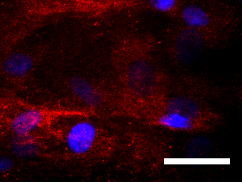


**Figure S7. Stem Cell Adhesion and Morphology on Plastic in Response to Stimulating Factors.** Human mesenchymal stem cells were cultured on plastic in the presence of the chondrogenic stimulator transforming growth factor-β (TGF-β). Following 3 days culture, cells were fluorescently stained for their focal adhesions (Paxillin, red) and cytoskeleton (Phalloidin, green) to assess the physical response of cells undergoing chondrogenesis under TGF-β stimulation with and without the use of blebbistatin. Cell nuclei are stained using DAPI (blue). *Scale bar inset measures 100* *µm, n=4. Higher magnification images shown for paxillin staining inset, scale bar measures 50 µm.*
